# A Review of Psychosocial Risk Factors for Pediatric Atopy

**DOI:** 10.1155/2012/821849

**Published:** 2012-03-05

**Authors:** Christina L. Duncan, Stacey L. Simon

**Affiliations:** ^1^Department of Psychology, West Virginia University, Morgantown, WV 26506-6040, USA; ^2^Division of Behavioral Medicine & Clinical Psychology, Cincinnati Children's Hospital Medical Center, Cincinnati, OH 45229-7036, USA

## Abstract

Pediatric atopy is increasing in prevalence and creates a significant financial and quality of life burden for children and families (e.g., frequent clinic visits, academic, and social challenges). Thus, it is important to understand modifiable risk factors related to disease onset or exacerbation in young children. The existing research base suggests that while a genetic link has been identified, specific family psychological factors (e.g., parent stress) also appear to play a significant role in the development of pediatric atopy. The function of psychological stress in the clinical expression and exacerbation of allergic diseases in young children is hypothesized to be due to neuroendocrine and immunologic systems. Specifically, stress-related activation of the sympathetic and adrenomedullary (SAM) system as well as the hypothalamic-pituitary-adrenocortical (HPA) axis from both the intrauterine environment and early childhood experiences may increase risk of childhood atopy above and beyond genetic risk. Consequently, prevention and intervention strategies aimed at reducing children's early exposure to stress and psychological difficulties in parents may prove beneficial in preventing or reducing the likelihood that their children will develop atopy.

## 1. Introduction

Pediatric atopy is increasingly prevalent and represents a significant financial and quality of life burden for children and families [1]. In addition to significant morbidity, effects of disease and its treatment, such as fatigue and discomfort as well as frequent clinic visits, may result in academic and social challenges for youth with atopy. Thus, it is extremely important to try to understand modifiable risk factors related to disease onset or exacerbation in young children. While a genetic link has been identified, the contributing role of family psychological factors, particularly parent stress, is beginning to be explored in the literature. This relationship is predominantly correlational in nature, and the mechanisms involved are largely unknown but have been hypothesized. The role of parent stress in relation to pediatric allergic sensitization or development of allergic disease in early childhood and a hypothesized underlying psychoneuroimmunological theory are discussed.

## 2. Parent-Report of Family Stress in Early Childhood

Early psychosocial factors can play a role in disease development later in childhood. In a cohort of youth, family stress early in life (ages 9–24 months) was associated with asthma status at 4 years of age [2]. However, parent stress was highly correlated with asthma symptom severity. Indeed, when both variables were considered, severity of asthma symptoms was a better predictor of asthma status than parent stress. Of note, at baseline participants in this study were already evidencing active wheezing, making it unclear whether parent stress preceded or resulted from asthma symptomatology. 

Most research has focused on mothers to the exclusion of paternal factors. As an exception to this methodological approach, both mothers and fathers in a cohort of Puerto Rican twins were interviewed about their psychological functioning during year one of their children's lives using the *Mood and Feelings Questionnaire* and the *World Health Organization Composite International Diagnostic Interview, Version 3.0 *[3]. Both maternal and paternal psychological symptoms were associated with negative health outcomes in early childhood. Specifically, paternal symptoms of PTSD, depression, and antisocial behavior were associated with recent asthma symptoms in youth at age one, including daytime and nighttime symptoms, and use of albuterol. For each additional parent endorsing depressive symptoms, there was a one-and-a-half time's increase in odds of a child having recent asthma symptoms. Of note, respiratory symptoms were elicited from parent self-report rather than confirmed diagnoses, and this study did not differentiate between symptoms that may have been present due to other reasons (e.g., respiratory tract infections). Additionally, the use of twins may limit the generalizability of the sample. Again, since parents were interviewed retrospectively, it is unable to be determined whether parental psychological symptoms led to asthma symptoms, or if the presence of these symptoms increased parent stress.

## 3. Maternal Report of Prenatal Stress

Examining parental stress during pregnancy and its relation to atopy development in early childhood can eliminate some of the confounding factors present when examining stress postnatally. The extant literature provides evidence that maternal stress during pregnancy can impact fetal development, with a variety of maladaptive responses including preterm birth, low birthweight, and increased susceptibility to chronic illness [4]. Utilizing a birth-cohort design, a large sample of infant-mother pairs recruited from hospitals in Taiwan was followed at 3rd trimester gestation as well as at 6 and 24 months of age [5]. Based on maternal self-report on the modified Chinese version of the *Short Form 36 Health Survey*, authors found that greater emotional stress endorsed during pregnancy, including symptoms of depression and anxiety, was associated with atopic dermatitis (AD) in early childhood [5]. A limiting factor of this study is use of parental report of atopic symptoms and purported physician diagnoses, which may have resulted in some youth's AD status being incorrectly classified.

## 4. Caregiver Stress Measured in Biomarkers

Eliminating a reliance on parental-report of youth atopic symptoms, Wright and colleagues [6] measured physiological markers of immune response in relation to caregiver stress. Their sample included 500 youth with a family history of asthma or allergy who were followed from birth. Caregiver stress was assessed bimonthly for the first two years of life and then yearly using the *Perceived Stress Scale* (PSS); biomarkers of atopy were obtained from blood samples two years later. Higher levels of caregiver stress in early childhood were associated with a corresponding change in atopic-specific biomarkers, including IgE and cytokine production at age 2-3 years.

## 5. Proposed Psychoneuroimmunological Theory Connecting Family Stress and Development of Pediatric Atopy

Taken together, the literature seems to support the notion that caregiver stress and parental psychological symptoms during pregnancy and early childhood are associated with increased risk of atopy. The role of psychological stress on clinical expression and exacerbation of allergic diseases in young children is hypothesized to be due to neuroendocrine and immunologic systems. Activation of the sympathetic and adrenomedullary (SAM) system as well as the hypothalamic-pituitary-adrenocortical (HPA) axis is documented to be associated with psychological stressors [7].

## 6. HPA Axis Dysregulation

The HPA axis is influential in regulating homeostasis. These systems may dysregulate in the presence of stressors, and this disturbance in homeostasis and resultant alterations in cortisol levels have been linked to development of disease processes. Effects may be both short- and long-term: the HPA axis is susceptible to early programming, thus prenatal stress may cause physiological dysregulation resulting in development of atopic symptoms, and these youth may be more sensitive to parental stress throughout childhood [8]. 

Youth cortisol levels also have been shown to be influenced by family stress. Salivary cortisol levels were measured in newborns with and without a genetic predisposition for atopy (e.g., one or more parents with a history of atopy) before and after a heel prick stress procedure [9]. Newborns with a parental history of atopy and elevated IgE (immunoglobulin-E) levels (a biomarker found to be related to atopy) demonstrated a hypercortisol response following the stressor. In contrast, other studies show a decreased cortisol response to stress with school-age youth [10] and adolescents [11] with atopic disorders. Thus, it may be that a genetic risk for atopy (e.g., parental history of atopy), combined with a tendency for HPA dysregulation, may increase risk of developing atopy later in life.

### 6.1. Epigenetic Influences

Indeed, there has been a line of animal and human research associating the development of atopy with epigenetic changes (i.e., permanent changes in gene function caused by environmental influences). Some research findings support the notion that the influence of prenatal and early life experience of psychosocial and physical stress is related to the development of atopic diseases in children [12]. Specifically, there has been growing evidence to support the theory that transitory family stress (e.g., maternal depression, paternal psychological dysfunction) can have permanent effects on gene regulation and expression in children via epigenetic mechanisms [12, 13]. For example, histone modifications have been linked to bronchial hyper-responsiveness and corticosteroid resistance in asthma [12]. Thus, it has been argued that the HPA axis is changed epigenetically by stress [14]. Taken together, it could be expected that these gene-environment changes in the HPA axis and its function could potentially affect the course of atopy as allergic rhinitis, asthma, and atopic dermatitis are all cortisol-responsive diseases.

## 7. Directions for Future Research

A link between family stress and mood and expression of atopic disease in youth is emerging in the literature, with the mechanism for this effect thought to be related to dysregulation of the HPA axis. Stress-related changes in SAM and HPA activity from both the intrauterine environment and early childhood may increase risk of childhood atopy above and beyond genetic risk. Given the negative financial and quality of life costs of childhood atopy, there is a vital need for ongoing research to better understand these modifiable risk factors.

To advance our understanding of the relation between psychosocial risk factors and the development of atopy in youth, it will be important for future research to improve upon the methodology of published studies. In particular, it will be essential for forthcoming research to utilize longitudinal designs, ideally beginning to gather psychosocial data with both parents early in pregnancy and following them and their children as the children age through preschool and into school. Atopic diagnoses should be confirmed by physicians or medical chart reviews, rather than obtained through parent report. Moreover, reliable and well-validated measures of psychological functioning should be used when measuring psychosocial risk factors. Finally, these studies need to be conducted in the United States, rather than specific to particular foreign countries, and should strive to include a sample that is broad in ethnic and racial characteristics. By doing so, the generalization of findings will be enhanced.

## 8. Clinical Applicability

Despite the need for increased research efforts, future research will need to investigate the impact of prevention and intervention strategies, as well as the potential benefits of psychopharmacological therapy. Some clinical implications can be derived from current findings. Specifically, the present literature suggests that prevention and intervention strategies aimed at reducing children's early exposure to stress and psychological difficulties in parents may prove beneficial in preventing or reducing the likelihood that their children will develop atopy. These strategies should be focused on parental stress management and mood symptom reduction via empirically validated approaches, such as cognitive behavioral therapy. At the very least, however, this paper suggests that pregnant women and new parents who report stress or psychological dysfunction likely would benefit from a referral for psychological services, and perhaps their young children will benefit as well. Thus, obstetricians and pediatricians should be aware of these potential clinical implications for the care of their patients.
